# The Durham County Hospital

**Published:** 1893-02-18

**Authors:** 


(BY OUR SPECIAL COMMISSIONER.)

BUILDINGS.

### THE DURHAM COUNTY HOSPITAL.

(BY OUR SPECIAL COMMISSIONER.)

This is a small, handsome, well-situated hospital, containing seventy beds. It is well maintained, and has an income more than sufficient to meet its expenditure. The hospital consists of a block of buildings, with a courtyard in the centre, the wards occupying the front of one side and the out-patient department the other side. The laundry, mortuary, and other buildings complete the square at the back. Well proportioned, and situate in front, are a number of small wards, the largest of which contains six beds. The later additions consist of a pavilion, occupying one side, of two storeys, containing two wards of good proportions, with admirable lavatory, bath, and other accommodation. We have no doubt that the patients find themselves in comfortable quarters when under treatment in the hospital, and the nursing and general management appear to be good. There is a resident medical officer, and he and the Matron seem devoted to their work, and could practically manage the whole institution. So far as we can gather, the residents of the City of Durham take unusually small personal interest in the work of the hospital, appearing to be content with the knowledge that it exists, that it contains patients, and that there are no complaints. Of the seventy beds, less than one-fourth were in daily occupation in the last six months of 1891. Sometimes only eight beds have been occupied at a time; the greatest number of in-patients at any one date in the same year was 43. We are sorry to observe that there has been a very large falling off in the contributions from the congregations on Hospital Sunday, a fact which we are at a loss to account for. It is satisfactory to know that the working classes chiefly use the hospital, and give an increasingly large contribution every year out of their earnings towards its support.

SAMARITAN SOCIETY.

A most important, if not the most important, portion of the work done at Durham seems to be undertaken by this society, which had an income of £457 in 1891. It appears to be devoted chiefly to the provision of district nurses, who attend the patients in their own homes, and supply them there, in addition to nursing, with diet, invalid appliances, and other surgical aids. In addition to this many patients are sent to convalescent homes, and stimulants are supplied where they may be necessary for the treatment of the case, and the patient is unable to provide them for himself. This Samaritan Society appears to be popular, as it deserves to be. The subscriptions and donations to it are considerable, while the residents, by sales of work and other movements, raise any balance of revenue which may be found necessary for the efficient conduct of this most useful work.

